# In-depth assessment of the PAM compatibility and editing activities of Cas9 variants

**DOI:** 10.1093/nar/gkab507

**Published:** 2021-06-16

**Authors:** Weiwei Zhang, Jianhang Yin, Zhengrong Zhang-Ding, Changchang Xin, Mengzhu Liu, Yuhong Wang, Chen Ai, Jiazhi Hu

**Affiliations:** The MOE Key Laboratory of Cell Proliferation and Differentiation, School of Life Sciences, Center for Life Sciences, Genome Editing Research Center, Peking University, Beijing 100871, China; The MOE Key Laboratory of Cell Proliferation and Differentiation, School of Life Sciences, Center for Life Sciences, Genome Editing Research Center, Peking University, Beijing 100871, China; The MOE Key Laboratory of Cell Proliferation and Differentiation, School of Life Sciences, Center for Life Sciences, Genome Editing Research Center, Peking University, Beijing 100871, China; The MOE Key Laboratory of Cell Proliferation and Differentiation, School of Life Sciences, Center for Life Sciences, Genome Editing Research Center, Peking University, Beijing 100871, China; The MOE Key Laboratory of Cell Proliferation and Differentiation, School of Life Sciences, Center for Life Sciences, Genome Editing Research Center, Peking University, Beijing 100871, China; The MOE Key Laboratory of Cell Proliferation and Differentiation, School of Life Sciences, Center for Life Sciences, Genome Editing Research Center, Peking University, Beijing 100871, China; The MOE Key Laboratory of Cell Proliferation and Differentiation, School of Life Sciences, Center for Life Sciences, Genome Editing Research Center, Peking University, Beijing 100871, China; The MOE Key Laboratory of Cell Proliferation and Differentiation, School of Life Sciences, Center for Life Sciences, Genome Editing Research Center, Peking University, Beijing 100871, China

## Abstract

A series of Cas9 variants have been developed to improve the editing fidelity or targeting range of CRISPR–Cas9. Here, we employ a high-throughput sequencing approach primer-extension-mediated sequencing to analyze the editing efficiency, specificity and protospacer adjacent motif (PAM) compatibility of a dozen of SpCas9 variants at multiple target sites in depth, and our findings validate the high fidelity or broad editing range of these SpCas9 variants. With regard to the PAM-flexible SpCas9 variants, we detect significantly increased levels of off-target activity and propose a trade-off between targeting range and editing specificity for them, especially for the near-PAM-less SpRY. Moreover, we use a deep learning model to verify the consistency and predictability of SpRY off-target sites. Furthermore, we combine high-fidelity SpCas9 variants with SpRY to generate three new SpCas9 variants with both high fidelity and broad editing range. Finally, we also find that the existing SpCas9 variants are not effective in suppressing genome instability elicited by CRISPR–Cas9 editing, raising an urgent issue to be addressed.

## INTRODUCTION

The antiviral system CRISPR–Cas9 of *Streptococcus pyogenes* bacterium has been engineered to be applied to different genome editing scenarios (1–6). The original CRISPR–Cas9 recognizes single guide RNA (sgRNA)-complementary 20-bp genomic sequences adjacent to an NGG protospacer adjacent motif (PAM). Similar to other sequence-specific endonucleases, CRISPR–Cas9 shows varied levels of genome-wide off-target activity at homologous sequences of the target sequences (7–10). A couple of high-fidelity *S. pyogenes* Cas9 (SpCas9) variants have been developed to enhance the discrimination of CRISPR–Cas9 on the off-target sites to reduce unintended damages (11–18). For instance, eSpCas9(1.1) and HF1 weaken the binding affinity between Cas9/sgRNA and DNA sequences to improve target specificity (11,12), while HypaCas9 enhances the proofreading capacity to improve CRISPR–Cas9 targeting accuracy (13). Moreover, some other high-fidelity SpCas9 variants have been developed via high-throughput screening assays, including evoCas9 and Sniper-Cas9 (14,15).

PAM contributes to the targeting specificity of CRISPR–Cas9 by adding extra essential nucleotides that are critical for Cas9 binding (19). However, PAM also limits the targeting scope of CRISPR–Cas9 as well as similar Cas-involved genome editing toolboxes. To broaden the targeting range, several PAM-flexible SpCas9 variants have been engineered. Cas9-NG, xCas9(3.7) and SpG require only NGN PAM compared to the original NGG for SpCas9 (20–22). The recently reported SpCas9 variant SpRY is even able to target DNA sequences bearing NNN PAMs, though exhibiting higher target activity at NRN than NYN (R for A or G, Y for C or T) (22). These PAM-flexible SpCas9 variants are especially useful for base editors that are often locus restricted (23).

To comprehensively evaluate the editing efficiency, targeting specificity, PAM compatibility and genome integrity of genome editing exerted by high-fidelity or PAM-flexible SpCas9 variants, we employed the high-throughput primer-extension-mediated sequencing (PEM-seq) (17) assay for in-depth analysis at target sites with different types of PAMs. We validate the activity of these SpCas9 variants and also find a trade-off between target efficiency and specificity for high-fidelity SpCas9 variants. We compared the targeting range of four PAM-flexible SpCas9 variants and used a deep learning model to investigate the off-target activity of the near-PAM-less SpRY. Moreover, we also uncovered the chromatin abnormality induced by these SpCas9 variants, which are invisible to previous analysis. Finally, we combined the high-fidelity and SpRY to generate several high-fidelity SpCas9 variants with a broad targeting range. This study gains more insight into the varied activity of high-fidelity and PAM-flexible SpCas9 variants and can shed light on further engineering of CRISPR–Cas9.

## MATERIALS AND METHODS

### Plasmid construction

For fair comparison among different SpCas9 variants, we generated all SpCas9 variants derived from the same parental SpCas9 based on the plasmid pX330 (Addgene 42230) backbone. SpCas9 variants were site-directed mutagenesis generated by Gibson assembly (New England Biolabs). The mutation information is shown in Supplementary Figures S1A and S6. All the plasmids have the same codon optimization, NLS configuration and a CMV-driven mCherry. sgRNA was cloned into another plasmid with a CMV-driven GFP. Sequence for sgRNA is shown in Supplementary Table S1.

### Cell culture and transfection

HEK293T cells were cultured in Dulbecco's modified Eagle's medium (Corning) with glutamine (Corning), 10% fetal bovine serum (FBS, Excell Bio) and penicillin/streptomycin (Corning) at 37°C under 5% CO_2_.

A total of 3 μg of the Cas9 plasmid and 3 μg of the sgRNA plasmid were co-transfected into 6-cm dish HEK293T cells by 18 μl of 1 mg/ml PEI (Sigma). Cells were harvested 72 h post-transfection and were sorted by fluorescence-activated cell sorting (FACS, MoFlo XDP, Beckman Coulter) according to mCherry and GFP followed by genomic DNA extraction.

### Cell lysis and genomic DNA extraction

After FACS, cells were washed with phosphate-buffered saline, then lysed by 500 μl lysis buffer [200 mM NaCl, 10 mM Tris–HCl (pH 7.4), 2 mM ethylenediaminetetraacetic acid (pH 8.0), 0.2% (wt/vol) sodium dodecyl sulfate, 200 ng/ml Proteinase K (Sigma)] and incubated at 56°C for 12 h. Then, 500 μl isopropanol was added to precipitate the genomic DNA (gDNA). The gDNA was dissolved into dH_2_O for PEM-seq operation.

### T7EI cleavage assay

General procedures were referred to the method described before (17). FastPfu (TransGen) DNA polymerase was used for general polymerase chain reaction (PCR) followed by purification, denaturation and reannealing of the PCR products. Then, T7EI (New England Biolabs) was used for digestion of the PCR products followed by electrophoresis. Primer sequence for each target site was listed in Supplementary Table S1.

### PEM-seq operation and analysis

PEM-seq construction and analysis for off-target, translocation and large deletion were referred to (17,24). Generally, biotinylated primer was designed within 150 bp around the Cas9-targeting site to achieve primer extension. Site-specific nested primer was designed for following amplification. All the PEM-seq libraries were sequenced by Illumina HiSeq. For off-target analysis, junctions proximal to break site (±20 kb) were excluded and *MACS2 callpeak* was used to identify translocation enriched region. Off-target hotspots were defined to have less than eight mismatches with on-target site and more than three junctions at the presumable cutting site. Translocations from general double-stranded breaks (DSBs) were calculated by excluding junctions ±20 kb around the target sites and ±100 bp around the off-target sites.

The primer sequence is shown in Supplementary Table. Plasmid insertion analysis was referred to (24).

### Deep learning for SpRY off-targets

General procedure is referred to (25). The input is a code matrix with shape of 23 (sgRNA and PAM) × 4 (A, T, C, G). The first layer is a convolutional layer, which is for extracting matching information. The second layer is a batch normalization layer, which is for reducing internal covariate shift in the neural network to speed up learning and avoid over-fitting. The third layer is a global max-pooling layer connected with the previous BN layer to call whether the mismatches modeled by the respective BN layer exist in the input sequence or not. The following layers are two dense layers which consist of 100 and 23 neurons, respectively. A dropout layer is used on the last dense layer to avoid over-fitting and the final output layer consists of one neuron using the sigmoid function. The input data for training are divided into two types: true off-targets detected by PEM-seq and false randomly generated sequences that has more than 10 mismatches with the target site, followed by 30 cycles of training. For the prediction, genomic sequences which have less than eight mismatches with target sequence were retrieved and subject to prediction.

### Statistical analysis

Wilcoxon-matched pairs singed rank test was used. *P*  <  0.05 was considered significant.

## RESULTS

### Activities of high-fidelity and PAM-flexible SpCas9 variants at NGG loci

**Figure 1.**
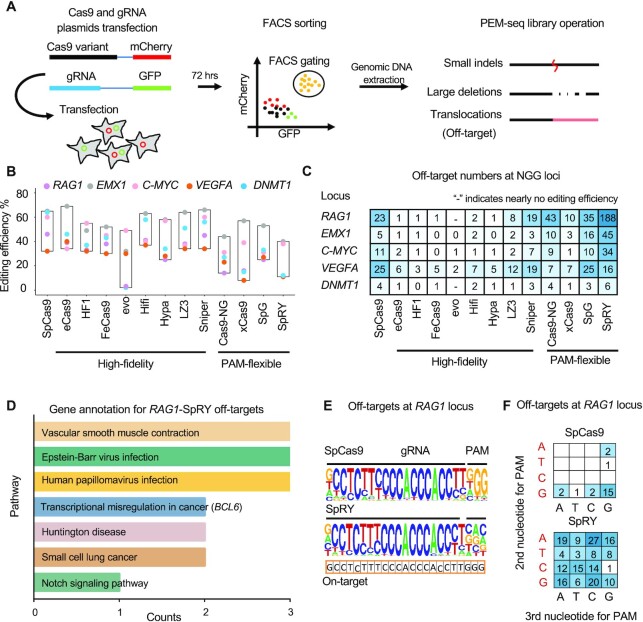
Evaluation for high-fidelity and PAM-flexible variants by PEM-seq at NGG loci. (**A**) Overview for SpCas9 variants' evaluation by PEM-seq. Plasmids carrying Cas9-mCherry and sgRNA-GFP were co-transfected into HEK293T cells followed by FACS and PEM-seq operation about 72 h later. PEM-seq can simultaneously detect small indels, large deletions and chromosomal translocations with off-target or general DSBs. (**B**) Editing efficiency for SpCas9 variants at indicated NGG loci detected by PEM-seq. Editing efficiency is referred to the total percentage of indels, large deletions and translocations. (**C**) Off-target numbers for SpCas9 variants at indicated NGG loci detected by PEM-seq. ‘-’ indicates nearly no editing activity (editing efficiency <2% is defined as nearly no editing activity). (**D**) Gene annotation for SpRY off-targets at *RAG1* locus using KEGG of Enrichr (maayanlab.cloud/Enrichr/). The horizontal axis indicates the gene numbers in the related pathways. (**E**) Consensus sequence analysis by weblogo (weblogo.berkeley.edu) for SpCas9 and SpRY off-targets at *RAG1* locus detected by PEM-seq. On-target sequence is marked below and position for sgRNA and PAM is labeled above. (**F**) Statistics for the second and third nucleotides of PAM for SpCas9 or SpRY off-targets at the *RAG1* locus detected by PEM-seq.

To extensively assess the editing activities of SpCas9 and SpCas9 variants, we employed the PEM-seq to capture various editing outcomes including small insertions/deletions (indels), large deletions and off-target translocations [Figure 1A, ref. (17,24) for technology details]. We selected eight high-fidelity SpCas9 variants (eCas9, HF1, FeCas9, evoCas9, Hypa, Hifi, LZ3 and Sniper) (11–18) and four PAM-flexible variants (Cas9-NG, xCas9, SpG and SpRY) (20–22) to target five conventional SpCas9-targeting sites with NGG PAMs within the *RAG1*, *EMX1*, *C-MYC*, *VEGFA* and *DNMT1* genes (Supplementary Figure S1A). All the variants were placed in the same plasmid backbone under the Chicken β-actin promoter and operated in parallel for a fair comparison. To collect edited genomic DNA for preparing PEM-seq libraries, we sorted the transduced HEK293T cells with Cas9-mCherry and sgRNA-GFP co-expression via FACS 72 h post-transfection (Figure 1A).

SpCas9 and all the tested high-fidelity SpCas9 variants were able to induce substantial cleavages at the five target sites except that evoCas9 showed almost undetectable cleavage activity at the *RAG1* and *DNMT1* sites (Figure 1B). The other high-fidelity SpCas9 variants showed comparable editing efficiencies at these sites with the SpCas9 despite some differences at certain sites for some variants (Figure 1B). As anticipated, all the high-fidelity variants showed generally significantly lower levels of off-target activities compared to the SpCas9 with LZ3 and Sniper being the least specific (Figure 1C). Moreover, the off-target sites identified by high-fidelity variants also occurred in the PEM-seq library of the SpCas9 as exemplified by the data from the *RAG1* target site (Supplementary Figure S1B and Table S1), indicating a similar targeting range of these variants with the SpCas9. A trade-off between editing efficiency and specificity was also found for high-fidelity SpCas9 variants (Supplementary Figure S1C), consistent with previous reports (18,26).

With regards to the PAM-flexible variants, the editing efficiencies at the tested NGG-PAM sites for the four variants were generally lower than the SpCas9 though still sufficient to induce efficient gene editing at the target sites (Figure 1B). Though fewer off-targets were detected in xCas9 samples, much more off-targets were found for Cas9-NG, SpG and especially for SpRY except at the *VEGFA* site with several very strong off-target sites harboring NGG PAMs (Figure 1C and Supplementary Table S1). For the *RAG1* site, a total of 188 off-targets were identified for SpRY and 109 of these off-targets lie in the genes involved in different molecular pathways including viral infection and cancers (27) (Figure 1D). Specifically, the *BCL6* gene, as one of the off-target, has been implicated in a variety of tumors, such as B-acute lymphoblastic leukemia and non-small cell lung cancer (28). Moreover, we sought to validate some top off-targets of SpRY at these NGG loci by T7EI assay. Though the sensitivity of T7EI is not as good as sequencing, cleavage was still detected at 8 out of 10 tested sites, except for the third off-target of *C-MYC* and the second off-target of *VEGFA* (Supplementary Figure S1D).

The consensus sequence of SpRY off-targets is relatively less conserved in the PAM-distal region of the sgRNA body, displaying a similar mismatch pattern to that of the SpCas9 (Figure 1E). Nonetheless, more off-targets of SpRY harbored higher numbers of mismatches than those from SpCas9 as exemplified by the *RAG1* and *EMX1* sites (Supplementary Figure S1E). The consensus PAM sequence for the off-targets of the SpCas9 resembled NGG, while SpRY showed no particular preferred nucleotide at the second or third position with a moderate bias of NRN against NYN (R for A or G, Y for C or T; Figure 1E), consistent with the initial report of SpRY (22). Collectively, broader PAM scope and higher tolerance of mismatch numbers lead to greatly increased off-target activity for SpRY. With regards to other variants, off-targets with NGN are favored by the xCas9, Cas9-NG and SpG, in line with their PAM preference (Supplementary Figure S1F) (20–22).

### Activities of PAM-flexible variants at NGH loci

**Figure 2.**
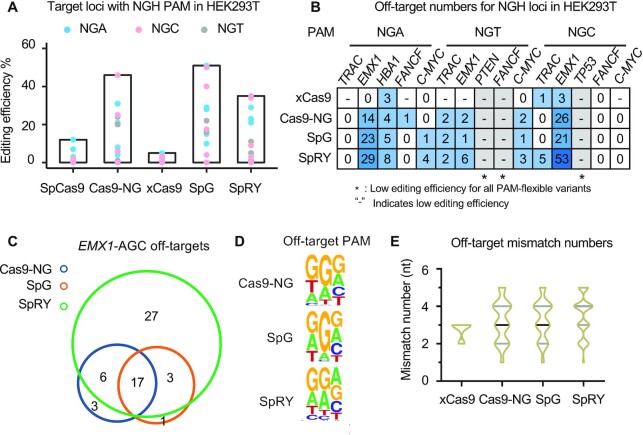
Comparison of SpCas9, Cas9-NG, xCas9, SpG and SpRY at NGH loci. (**A**) Editing efficiency for SpCas9 and indicated PAM-flexible variants at 15 NGH (NGA, NGC or NGT) loci in HEK293T cells detected by PEM-seq. (**B**) Off-target numbers at indicated NGH loci detected by PEM-seq. ‘-’ indicates nearly no editing activity. * indicates low editing efficiency for all variants. PAM and locus information are marked above. (**C**) Venn diagram for off-targets of Cas9-NG, SpG and SpRY at *EMX1*-AGC locus detected by PEM-seq. (**D**) Consensus analysis by weblogo for PAM sequence for Cas9-NG, SpG and SpRY off-targets for all NGH loci detected by PEM-seq. (**E**) Statistics of sgRNA mismatch numbers between off-target and on target for all 15 NGH loci for indicated SpCas9 variants detected by PEM-seq.

To further assess the PAM compatibility of these PAM-flexible SpCas9 variants at NGH PAMs (NGA, NGT, or NGC) in human cells, we designed five target sites for each type of PAM at genes, including *TRAC*, *EMX1*, *HBA1*, *FANCF* and *C-MYC*. We then used PEM-seq for in-depth analysis of CRISPR editing at these target loci in the HEK293T cells. The SpCas9 only exhibited detectable cleavage activity at the target sites with NGA PAM (Figure 2A), in line with previous reports that the NGA is also targetable by CRISPR–Cas9 (29). The Cas9-NG, SpG and SpRY showed robust editing activity at most target sites except two NGT sites in *PTEN* and *FANCF* genes in addition to an NGC site in the *TP53* gene; however, xCas9 showed the lowest editing capacity and the cleavage was almost undetectable at most tested sites regardless of the PAM composition (Figure 2A). Correspondingly, we detected off-targets from several to tens for these PAM-flexible variants at tested sites and SpRY universally cleaved at more off-target sites than the other variants (Figure 2B and Supplementary Table S1). Moreover, most of the identified off-targets are shared by Cas9-NG, SpG and SpRY (Figure 2C). The occurrences of several unique off-targets for Cas9-NG and SpG are probably due to compatible but minorly different preference at the NGH PAMs that the SpG showed the strictest constraint at the second G than Cas9-NG and then SpRY (Figure 2D; examples in Supplementary Figure S2A and B). With regards to mismatch at the sgRNA sequences, the tolerance from high to low is in an order of SpRY > Cas9-NG ≈ SpG > xCas9 with similar general mismatch patterns (Figure 2E; Supplementary Figure S2A and B), in line with the above findings at target sites with NGG PAMs.

### Activities of PAM-less SpRY at NHN loci

**Figure 3.**
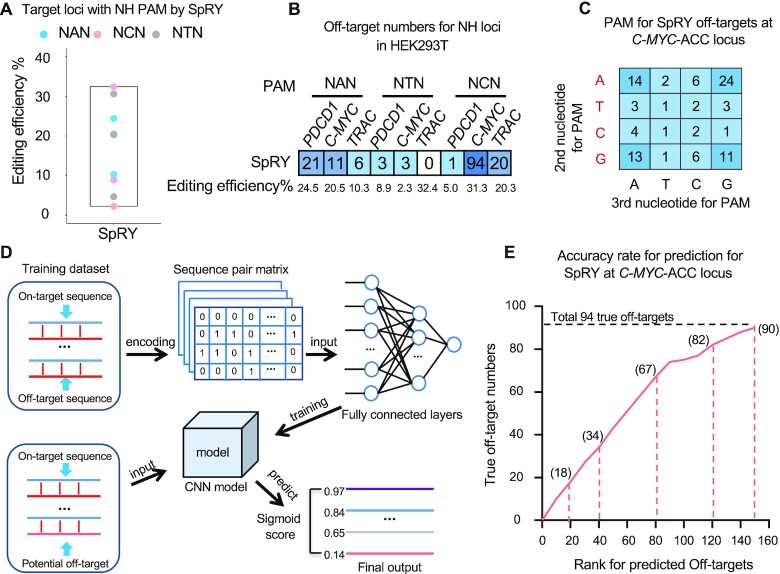
Evaluation for SpRY at NH loci. (**A**) Editing efficiency for SpRY at indicated NH loci detected by PEM-seq. (**B**) Off-target numbers at indicated NH loci detected by PEM-seq. Corresponding editing efficiencies are indicated at the bottom. (**C**) Statistics for the second and third nucleotides of PAM for SpRY off-targets at *C-MYC*-ACC locus detected by PEM-seq. (**D**) Overview for deep learning procedures for SpRY off-target prediction. Sequences for off-targets paired with on-target converted into matrix were used for training CNN model. For predictions, genome-wide 23-nt sequences less than eight mismatches with the on-target were subject to prediction. The predicted off-targets are listed by Sigmoid score. See more details in the ‘Materials and Methods’ section. (**E**) Off-target prediction by CNN for *C-MYC*-ACC locus. The horizontal axis indicates the rank for off-targets predicted by CNN model. The vertical axis indicates true numbers of off-targets in the rank. Corresponding true off-target numbers are labeled. Total 94 true off-targets are indicated by the black dashed line.

SpRY is currently the only near PAM-less SpCas9 variant and greatly broadens the targeting range of CRISPR–Cas9. To assess the activities of SpRY at NHN PAMs (NAN, NCN, or NTN), we designed three target sites for each type of PAM in HEK293T cells and employed PEM-seq for in-depth analysis. Overall, SpRY showed varied editing cleavage, ranging from 2.3 to 32.4% at these loci (Figure 3A). Several to almost one hundred off-target sites were detected for these target loci except none for the *TRAC* site with an NTN PAM (Figure 3B and Supplementary Table S1). These off-target PAMs predispose to NNN with a minor bias of R (A or G) at the second position as anticipated (Figure 3C; Supplementary Figure S3A and B). For example, 77 off-targets have NRN PAMs while 17 with NYN PAMs at the *C-MYC*-ACC target site (Figure 3C).

As our data revealed a trade-off between editing range and targeting specificity for SpRY, we adapted a deep learning model developed for evaluating CRISPR–Cas9 off-targets (25) to test the consistency of SpRY off-targets among different tested sites and thereby for further off-target prediction. We collected the 23-bp information (sgRNA + PAM) from a total of 456 off-targets from our SpRY PEM-seq data to train the convolutional neural networks (CNN)-based model (Figure 3D) and saved the *C-MYC*-ACC site (from Figure 3C) for prediction. The ‘accuracy’ and ‘loss’ of the learning model achieved 97.8 and 7.5% after data learning of 10 epochs and finally reached 99.5 and 2.0%, respectively (Supplementary Figure S3C). For the prediction, we retrieved the *C-MYC*-ACC target-site-similar sequences within eight mismatches from the human hg38 genome and subjected them to the trained model for prediction. All the top 15 and 67/80 predicted sites are true off-targets as validated by the PEM-seq data and 90/94 identified off-targets occur in the top 150 predicted sites (Figure 3E; Supplementary Figure S3D and Table S1), indicating a decent performance of the trained deep learning model for SpRY off-target prediction.

### Genome instability during genome editing via CRISPR–Cas9 variants

**Figure 4.**
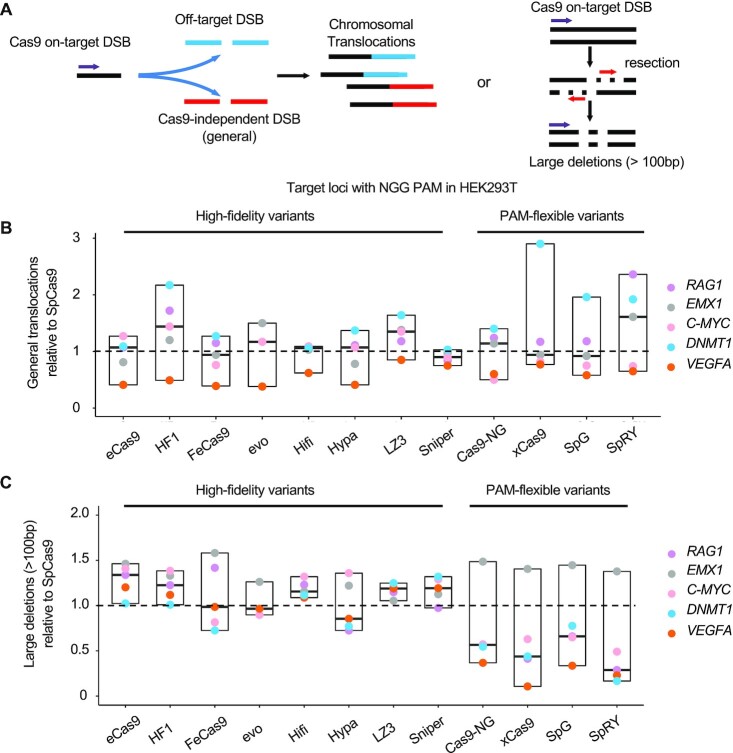
Genome instability caused by SpCas9 variants. (**A**) Schematics for the generation of translocations and large deletions caused by Cas9 cleavage. Cas9 on-target DSB can form translocations with off-target DSBs or Cas9-independent (general) DSBs. Large deletions (>100 bp) are caused by Cas9 cleavage followed by DNA resection. Red arrows indicate the 5′ to 3′ DNA resection orientations. Purple arrows indicate primers for PEM-seq. (**B** and **C**) Relative ratio of translocations (**B**) and large deletions (**C**) caused by SpCas9 variants normalized to the SpCas9 for indicated loci in HEK293T cells detected by PEM-seq.

The DNA repair outcomes induced by CRISPR–Cas9-activated DNA repair pathways have raised great concerns recently (17,30–33). Among these DNA repair outcomes, chromatin abnormality caused by large deletions (>100 bp) and chromosomal translocations is the most dangerous. Therefore, we used the levels of large deletions and translocations to represent genome instability elicited by genome editing as previously described (Figure 4A) (24). In order to detect chromatin abnormality for all the SpCas9 variants, we analyzed the PEM-seq data from CRISPR editing at five target sites with NGG PAMs. For the SpCas9, large deletions and translocations occur at average rates of 3.2 and 6.2%, respectively (Supplementary Figure S4A and B). Though showing great potential in reducing the off-target activity of SpCas9, the high-fidelity variants displayed comparable levels of chromosomal translocations as well as large deletions at tested sites (Figure 4B and C; Supplementary Figure S4A and B). With regards to the PAM-flexible variants, elevated levels of translocations were detected at *RAG1* (1.5-fold) and *DNMT1* (2.0-fold) sites due to more translocations between the target sites and off-target sites, while similar levels were detected for the *EMX1* and *C-MYC* sites (Figure 4B and Supplementary Figure S4A). Reduced levels of large deletions (2-fold on average) were detected for these PAM-flexible variants except at the *EMX1* site (Figure 4C and Supplementary Figure S4B). Unfortunately, these data suggested that the current high-fidelity or PAM-flexible SpCas9 variants are not able to suppress genome instability during genome editing, the same problem as the SpCas9.

### Plasmid integrations during genome editing via PAM-flexible SpCas9 variants

**Figure 5.**
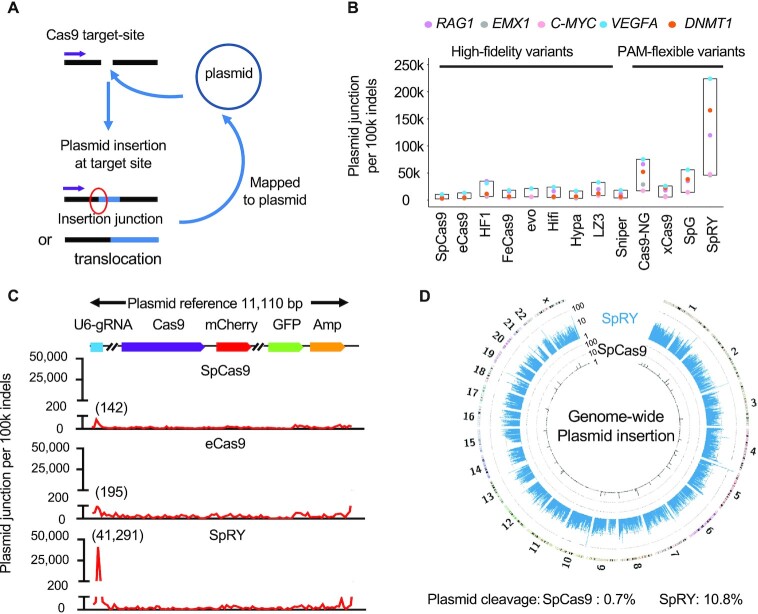
Plasmid cleavage for SpCas9 PAM-flexible variants. (**A**) Overview for the identification for plasmid junctions by PEM-seq. Purple arrows indicate the primers placed on the Cas9 target site in the genome. Plasmid junctions can be divided into two types dependent on the sequenced lengths: insertion or translocation. (**B**) Plasmid junctions per 100k on-target indels for SpCas9 variants at indicated NGG loci in HEK293T cells detected by PEM-seq cloning from the genomic loci. k for thousand. (**C**) The distribution of plasmid junctions across the plasmid backbone every 100k indels for SpCas9, eCas9 and SpRY at *C-MYC* locus in HEK293T cells detected by PEM-seq. Junction numbers for the U6-sgRNA region are marked above. The plasmid reference is on the top. Binsize = 100 bp. (**D**) Circos plot for the SpCas9 and SpRY libraries at *C-MYC* locus cloning from the plasmid. Translocation junctions for SpRY and SpCas9 are displayed from outside to inside, with numbers at 15 767 and 669, respectively. Genome-wide translocation junctions binned into 2-Mb regions (SpCas9: black lines and SpRY: blue lines) are plotted on a log scale. Density is labeled. Percentages of indels in the plasmid are marked on the bottom.

Plasmid integrations have been widely observed during CRISPR–Cas9 genome editing with DNA-based delivery systems including adeno-associated virus (AAV) and plasmids (24,34,35). To detect plasmid integrations for these SpCas9 variants, we analyzed the PEM-seq data as previously described (Figure 5A) (24). We found low levels of plasmid integrations for the SpCas9 and high-fidelity variants at the five tested sites with NGG PAMs and the inserted plasmid fragments were evenly distributed across the plasmid backbone (Figure 5B and C; Supplementary Figure S5A). The three PAM-flexible variants Cas9-NG, SpG and SpRY exhibited elevated levels of plasmid integrations when targeting at the five NGG target sites (SpRY > Cas9–NG> SpG) with significant enrichments at the U6-sgRNA regions compared to the SpCas9 (Figure 5B and C; Supplementary Figure S5A). For SpRY, we found 41 291 plasmid integrations per 100k editing events in the U6-sgRNA region, about 300-fold higher than that of the SpCas9 (Figure 5B and C; Supplementary Figure S5A). Though the total levels of plasmid integrations are not increased significantly for xCas9, enrichment at the U6-sgRNA regions is still detected (Figure 5B and Supplementary Figure S5A). In a zoomed-in view of SpRY, the enrichments mainly occur around the N_17_ and N_18_ of the sgRNA body CACC (N)_20_ GTTT, suggesting potential SpRY cleavage at the plasmids (Supplementary Figure S5B), consistent with a previous report in plants (36).

To verify the cleavage of SpRY at plasmids, we generated a PEM-seq library from a primer lying 53-bp downstream of the sgRNA in the plasmid to detected indels within the plasmids as well as plasmid-genome fusions. About 10% of plasmids were cleaved by SpRY calculated from the PEM-seq data (Figure 5D). Substantial plasmid-genome fusion junctions were detected and distributed widely in the genome in the SpRY-edited HEK293T cells (Figure 5D). Due to the lack of the NGG PAM, the SpCas9 is not supposed to cleave at the plasmid, and only background level of indels (0.7%) was detected (Figure 5D). Moreover, we placed a Cas9-target site in the plasmid to induce dual cleavage at both plasmid and the genome and finally detected a large number of plasmid-genome fusion junctions, providing further evidence for the danger of using targetable plasmid or virus for SpCas9 or variants delivery (Supplementary Figure S5C).

### Enhancing the targeting specificity of SpRY

**Figure 6.**
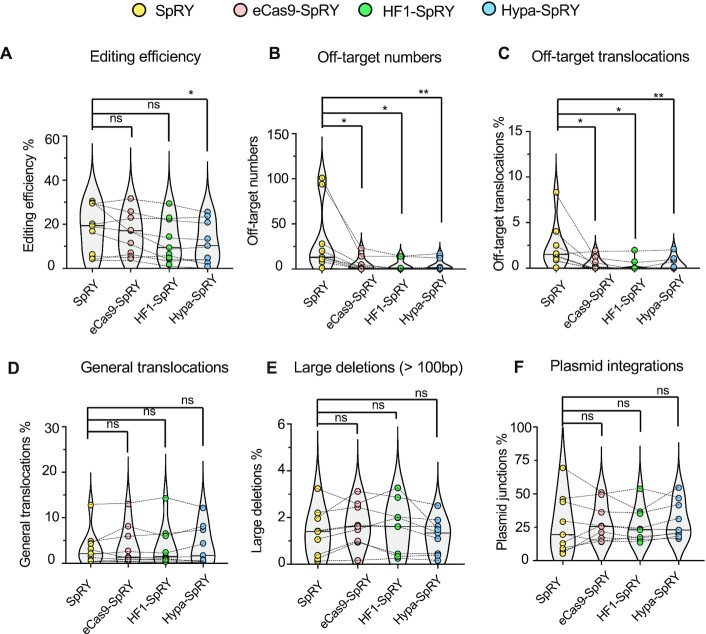
Enhancing SpRY specificity by combining with high-fidelity variants. Editing efficiencies (**A**), off-target numbers (**B**), percentages of off-target translocations (**C**), percentages of general translocations (**D**), large deletions (>100 bp) (**E**) and plasmid integrations (**F**) for SpRY, eCas9-SpRY, HF1-SpRY and Hypa-SpRY at indicated loci in HEK293T cells detected by PEM-seq. *N* = 9, loci are *PDCD1*-ACC or CAG, *C-MYC*-ACC or GGG, *RAG1*-GGG, *EMX1*-GGG, *TRAC*-ACT, *HBA1*-AGA and *VEGFA*-TGG. Wilcoxon matched-pairs signed rank test: *P* < 0.05 means significant.

The combination of SpRY with high-fidelity variant mutations may help improve the specificity of SpRY. To this end, we introduced the mutations of the three best high-fidelity variants eCas9, HF1 and HypaCas9 into the gene of *SpRY* to generate the eCas9-SpRY, HF1-SpRY and Hypa-SpRY (Supplementary Figure S6A). We applied PEM-seq for evaluating these combined SpCas9 variants at nine tested loci with the most off-targets. These sites harbored NGG, AGA, CAG, ACC or ACT PAMs. Compared to SpRY, eCas9-SpRY and HF1-SpRY showed comparable editing efficiencies at tested loci, while slightly lower editing efficiency for Hypa-SpRY (Figure 6A). The numbers of identified off-target sites for all the three combined variants at the nine tested sites are decreased significantly and the off-targets were even undetectable at several loci for HF1-SpRY and Hypa-SpRY (Figure 6B). Correspondingly, the levels of translocation events between on-target and off-target sites were also reduced significantly (Figure 6C and Supplementary Table S1), indicating a great improvement for specificity. However, similar or elevated levels of chromosomal translocations, large deletions and plasmid integrations were detected for eCas9-SpRY, HF1-SpRY and Hypa-SpRY versus SpRY (Figure 6D–F), indicating high levels of genome instability with these SpRY-based Cas9 variants.

## DISCUSSION

Both high-fidelity and PAM-flexible SpCas9 variants have been evaluated previously by other research groups (18,26,37,38). Whereas the previous assessments utilize a multiplexing system with tens of thousands of parallel target sites in the same library in order to cover as many as different types of SpCas9 variant-targeting sites in the genome (18,26,37), here we used a complementary strategy to assess the PAM compatibility, editing efficiency and targeting specificity of these SpCas9 variants by in-depth analysis of editing outcomes at multiple typical target sites with PEM-seq. Our strategy confirms the main findings in the previous studies while also brings new findings of the heterogeneity and complexity of gene editing behaviors of these SpCas9 variants. For instance, SpRY shows 188 off-targets in the *RAG1* site with an NGG PAM while none at some other sites including the *TRAC*-NGA and the *TRAC*-NTN site (Figure 1C, 2B and 3B). Moreover, large deletions and general translocations fused by the on-target and genome-wide general DSBs were constant among SpCas9 and its high-fidelity variants (Figure 4B and C) or SpRY and its high-fidelity variants (Figure 6D and F). These findings can be explained by that large deletions and general translocations are determined by DSB repair pathways and these variants are supposed to have no significant impact on the choice of DSB repair pathways.

The in-depth analysis shows the efficacy of using high-fidelity SpCas9 variants to reduce off-target activity and using PAM-flexible SpCas9 variants to broaden the editing range of CRISPR–Cas9 in the genome. However, the PAM compatibility of PAM-flexible SpCas9 variants, especially of SpRY, has been improved for both on-target and off-target activity (e.g. Figure 1F), which may lead to elevated levels of off-target damages. The mismatch patterns in the sgRNA body of these SpRY off-targets are similar to the SpCas9 (Figure1E). Besides, the utilization of PAM for SpRY on- and off-targets also has some features remaining to be explored, e.g. A/G bias. In this context, we used a deep learning model (25) to verify the consistency of these SpRY off-targets, which should be improved when feeding the CNN-based model with more data. The combination of SpRY with high-fidelity variants including eCas9, HF1 and HypaCas9 can largely improve the fidelity of SpRY and make it feasible for some genome editing scenarios.

High levels of plasmid integrations have been detected for these PAM-flexible SpCas9 variants, especially for the PAM-less SpRY, due to potential cleavage of SpCas9 variants at the plasmids (Figure 5). In this context, the DNA-based delivery systems, including the AAV, are not applicable for transducing PAM-flexible SpCas9 variants into cells. This is not limited to the Cas9 forms of these variants but also includes derived base editors, since base editors may also generate substantial mutations on the sgRNA sequence in the plasmids. Ribonucleoprotein (RNP) would be an optimal choice currently. Further optimization is in demand to suppress plasmid attacking of PAM-flexible SpCas9 variants as well as genome instability induced by SpCas9 or these SpCas9 variants. Moreover, since the editing outcomes can be affected by different transfection methods (DNA-based, RNA-based, RNP), further studies are needed to compare these variants using mRNA or RNP transfection.

## DATA AVAILABILITY

Data were deposited on NODE (National Omics Data Encyclopedia) database: OEP001824. Scripts and raw data of off-target prediction via deep learning model in this study are available at GitHub repository (https://github.com/JiazhiHuLab/CNN_predict) (25).

## Supplementary Material

gkab507_Supplemental_Files

## References

[1] Jinek M., Chylinski K., Fonfara I., Hauer M., Doudna J.A., Charpentier E. A programmable dual-RNA-guided DNA endonuclease in adaptive bacterial immunity. Science. 2012; 337:816–821. 22745249 10.1126/science.1225829 PMC6286148

[2] Cong L., Ran F.A., Cox D., Lin S., Barretto R., Habib N., Hsu P.D., Wu X., Jiang W., Marraffini L.A. et al. Multiplex genome engineering using CRISPR/Cas systems. Science. 2013; 339:819–823. 23287718 10.1126/science.1231143 PMC3795411

[3] Jinek M., East A., Cheng A., Lin S., Ma E., Doudna J. RNA-programmed genome editing in human cells. eLife. 2013; 2:e00471. 23386978 10.7554/eLife.00471 PMC3557905

[4] Mali P., Yang L., Esvelt K.M., Aach J., Guell M., DiCarlo J.E., Norville J.E., Church G.M. RNA-guided human genome engineering via Cas9. Science. 2013; 339:823–826. 23287722 10.1126/science.1232033 PMC3712628

[5] Zhang F. Development of CRISPR–Cas systems for genome editing and beyond. Q. Rev. Biophys. 2019; 52:e6.

[6] Doudna J.A. The promise and challenge of therapeutic genome editing. Nature. 2020; 578:229–236. 32051598 10.1038/s41586-020-1978-5 PMC8992613

[7] Frock R.L., Hu J., Meyers R.M., Ho Y.J., Kii E., Alt F.W. Genome-wide detection of DNA double-stranded breaks induced by engineered nucleases. Nat. Biotechnol. 2015; 33:179–186. 25503383 10.1038/nbt.3101 PMC4320661

[8] Kim D., Bae S., Park J., Kim E., Kim S., Yu H.R., Hwang J., Kim J.I., Kim J.S. Digenome-seq: genome-wide profiling of CRISPR–Cas9 off-target effects in human cells. Nat. Methods. 2015; 12:237–243. 25664545 10.1038/nmeth.3284

[9] Tsai S.Q., Zheng Z., Nguyen N.T., Liebers M., Topkar V.V., Thapar V., Wyvekens N., Khayter C., Iafrate A.J., Le L.P. et al. GUIDE-seq enables genome-wide profiling of off-target cleavage by CRISPR–Cas nucleases. Nat. Biotechnol. 2015; 33:187–197. 25513782 10.1038/nbt.3117 PMC4320685

[10] Cho S.W., Kim S., Kim Y., Kweon J., Kim H.S., Bae S., Kim J.S. Analysis of off-target effects of CRISPR/Cas-derived RNA-guided endonucleases and nickases. Genome Res. 2014; 24:132–141. 24253446 10.1101/gr.162339.113 PMC3875854

[11] Kleinstiver B.P., Pattanayak V., Prew M.S., Tsai S.Q., Nguyen N.T., Zheng Z., Joung J.K. High-fidelity CRISPR–Cas9 nucleases with no detectable genome-wide off-target effects. Nature. 2016; 529:490–495. 26735016 10.1038/nature16526 PMC4851738

[12] Slaymaker I.M., Gao L., Zetsche B., Scott D.A., Yan W.X., Zhang F. Rationally engineered Cas9 nucleases with improved specificity. Science. 2016; 351:84–88. 26628643 10.1126/science.aad5227 PMC4714946

[13] Chen J.S., Dagdas Y.S., Kleinstiver B.P., Welch M.M., Sousa A.A., Harrington L.B., Sternberg S.H., Joung J.K., Yildiz A., Doudna J.A. Enhanced proofreading governs CRISPR–Cas9 targeting accuracy. Nature. 2017; 550:407–410. 28931002 10.1038/nature24268 PMC5918688

[14] Casini A., Olivieri M., Petris G., Montagna C., Reginato G., Maule G., Lorenzin F., Prandi D., Romanel A., Demichelis F. et al. A highly specific SpCas9 variant is identified by *in vivo* screening in yeast. Nat. Biotechnol. 2018; 36:265–271. 29431739 10.1038/nbt.4066 PMC6066108

[15] Lee J.K., Jeong E., Lee J., Jung M., Shin E., Kim Y.H., Lee K., Jung I., Kim D., Kim S. et al. Directed evolution of CRISPR–Cas9 to increase its specificity. Nat. Commun. 2018; 9:3048. 30082838 10.1038/s41467-018-05477-x PMC6078992

[16] Vakulskas C.A., Dever D.P., Rettig G.R., Turk R., Jacobi A.M., Collingwood M.A., Bode N.M., McNeill M.S., Yan S., Camarena J. et al. A high-fidelity Cas9 mutant delivered as a ribonucleoprotein complex enables efficient gene editing in human hematopoietic stem and progenitor cells. Nat. Med. 2018; 24:1216–1224. 30082871 10.1038/s41591-018-0137-0 PMC6107069

[17] Yin J., Liu M., Liu Y., Wu J., Gan T., Zhang W., Li Y., Zhou Y., Hu J. Optimizing genome editing strategy by primer-extension-mediated sequencing. Cell Discov. 2019; 5:18. 30937179 10.1038/s41421-019-0088-8 PMC6434046

[18] Schmid-Burgk J.L., Gao L., Li D., Gardner Z., Strecker J., Lash B., Zhang F. Highly parallel profiling of Cas9 variant specificity. Mol. Cell. 2020; 78:794–800. 32187529 10.1016/j.molcel.2020.02.023 PMC7370240

[19] Hille F., Richter H., Wong S.P., Bratovic M., Ressel S., Charpentier E. The biology of CRISPR-Cas: backward and forward. Cell. 2018; 172:1239–1259. 29522745 10.1016/j.cell.2017.11.032

[20] Hu J.H., Miller S.M., Geurts M.H., Tang W., Chen L., Sun N., Zeina C.M., Gao X., Rees H.A., Lin Z. et al. Evolved Cas9 variants with broad PAM compatibility and high DNA specificity. Nature. 2018; 556:57–63. 29512652 10.1038/nature26155 PMC5951633

[21] Nishimasu H., Shi X., Ishiguro S., Gao L., Hirano S., Okazaki S., Noda T., Abudayyeh O.O., Gootenberg J.S., Mori H. et al. Engineered CRISPR–Cas9 nuclease with expanded targeting space. Science. 2018; 361:1259–1262. 30166441 10.1126/science.aas9129 PMC6368452

[22] Walton R.T., Christie K.A., Whittaker M.N., Kleinstiver B.P. Unconstrained genome targeting with near-PAMless engineered CRISPR–Cas9 variants. Science. 2020; 368:290–296. 32217751 10.1126/science.aba8853 PMC7297043

[23] Anzalone A.V., Koblan L.W., Liu D.R. Genome editing with CRISPR-Cas nucleases, base editors, transposases and prime editors. Nat. Biotechnol. 2020; 38:824–844. 32572269 10.1038/s41587-020-0561-9

[24] Liu M., Zhang W., Xin C., Yin J., Shang Y., Ai C., Li J., Meng F., Hu J. Global detection of DNA repair outcomes induced by CRISPR–Cas9. 2021; bioRxiv doi:16 February 2021, preprint: not peer reviewed 10.1101/2021.02.15.431335. PMC8421148 34365511

[25] Lin J., Wong K.C. Off-target predictions in CRISPR–Cas9 gene editing using deep learning. Bioinformatics. 2018; 34:i656–i663. 30423072 10.1093/bioinformatics/bty554 PMC6129261

[26] Kim N., Kim H.K., Lee S., Seo J.H., Choi J.W., Park J., Min S., Yoon S., Cho S.R., Kim H.H. Prediction of the sequence-specific cleavage activity of Cas9 variants. Nat. Biotechnol. 2020; 38:1328–1336. 32514125 10.1038/s41587-020-0537-9

[27] Kuleshov M.V., Jones M.R., Rouillard A.D., Fernandez N.F., Duan Q., Wang Z., Koplev S., Jenkins S.L., Jagodnik K.M., Lachmann A. et al. Enrichr: a comprehensive gene set enrichment analysis web server 2016 update. Nucleic Acids Res. 2016; 44:W90–W97. 27141961 10.1093/nar/gkw377 PMC4987924

[28] Cardenas M.G., Oswald E., Yu W., Xue F., MacKerell A.D. Jr, Melnick A.M. The expanding role of the BCL6 oncoprotein as a cancer therapeutic target. Clin. Cancer Res. 2017; 23:885–893. 27881582 10.1158/1078-0432.CCR-16-2071 PMC5315622

[29] Zhang Y., Ge X., Yang F., Zhang L., Zheng J., Tan X., Jin Z.B., Qu J., Gu F. Comparison of non-canonical PAMs for CRISPR/Cas9-mediated DNA cleavage in human cells. Sci. Rep. 2014; 4:5405. 24956376 10.1038/srep05405 PMC4066725

[30] Shin H.Y., Wang C., Lee H.K., Yoo K.H., Zeng X., Kuhns T., Yang C.M., Mohr T., Liu C., Hennighausen L. CRISPR/Cas9 targeting events cause complex deletions and insertions at 17 sites in the mouse genome. Nat. Commun. 2017; 8:15464. 28561021 10.1038/ncomms15464 PMC5460021

[31] Adikusuma F., Piltz S., Corbett M.A., Turvey M., McColl S.R., Helbig K.J., Beard M.R., Hughes J., Pomerantz R.T., Thomas P.Q. Large deletions induced by Cas9 cleavage. Nature. 2018; 560:E8–E9. 30089922 10.1038/s41586-018-0380-z

[32] Kosicki M., Tomberg K., Bradley A. Repair of double-strand breaks induced by CRISPR–Cas9 leads to large deletions and complex rearrangements. Nat. Biotechnol. 2018; 36:765–771. 30010673 10.1038/nbt.4192 PMC6390938

[33] Cullot G., Boutin J., Toutain J., Prat F., Pennamen P., Rooryck C., Teichmann M., Rousseau E., Lamrissi-Garcia I., Guyonnet-Duperat V. et al. CRISPR–Cas9 genome editing induces megabase-scale chromosomal truncations. Nat. Commun. 2019; 10:1136. 30850590 10.1038/s41467-019-09006-2 PMC6408493

[34] Hanlon K.S., Kleinstiver B.P., Garcia S.P., Zaborowski M.P., Volak A., Spirig S.E., Muller A., Sousa A.A., Tsai S.Q., Bengtsson N.E. et al. High levels of AAV vector integration into CRISPR-induced DNA breaks. Nat. Commun. 2019; 10:4439. 31570731 10.1038/s41467-019-12449-2 PMC6769011

[35] Norris A.L., Lee S.S., Greenlees K.J., Tadesse D.A., Miller M.F., Lombardi H.A. Template plasmid integration in germline genome-edited cattle. Nat. Biotechnol. 2020; 38:163–164. 32034391 10.1038/s41587-019-0394-6

[36] Ren Q., Sretenovic S., Liu S., Tang X., Huang L., He Y., Liu L., Guo Y., Zhong Z., Liu G. et al. PAM-less plant genome editing using a CRISPR-SpRY toolbox. Nat. Plants. 2021; 7:25–33. 33398158 10.1038/s41477-020-00827-4

[37] Kim H.K., Lee S., Kim Y., Park J., Min S., Choi J.W., Huang T.P., Yoon S., Liu D.R., Kim H.H. High-throughput analysis of the activities of xCas9, SpCas9-NG and SpCas9 at matched and mismatched target sequences in human cells. Nat. Biomed. Eng. 2020; 4:111–124. 31937939 10.1038/s41551-019-0505-1

[38] Legut M., Daniloski Z., Xue X., McKenzie D., Guo X., Wessels H.H., Sanjana N.E. High-throughput screens of PAM-flexible Cas9 variants for gene knockout and transcriptional modulation. Cell Rep. 2020; 30:2859–2868. 32130891 10.1016/j.celrep.2020.02.010 PMC7558435

