# Supplementary material for: In-depth assessment of the PAM compatibility and editing activities of Cas9 variants
Source: Nucleic Acids Res. 2021 Jun 16;49(15):8785–95. doi: 10.1093/nar/gkab507 (PMC8421146; doi:10.1093/nar/gkab507)
Supplement: gkab507_Supplemental_Files [file gkab507_supplemental_files.zip › Supplementary Figure.pdf]

Supplementary Materials for

In-depth assessment of the PAM compatibility and editing activities of Cas9 variants

Weiwei Zhang[1, 2], Jianhang Yin[1, 2,*], Zhengrong Zhang-Ding[1], Changchang Xin[1], Mengzhu Liu[1], Yuhong Wang[1], Chen Ai[1], Jiazhi Hu[1,*]

[1]The MOE Key Laboratory of Cell Proliferation and Differentiation, School of Life Sciences, Center for Life Sciences, Genome Editing Research Center, Peking University, Beijing 100871, China

[2]These authors contributed equally to this work.

*Corresponding author: yjh2016@pku.edu.cn and hujz@pku.edu.cn

This PDF file includes:

Supplementary Figure 1. Evaluation of the editing efficiency and specificity for 8 high-fidelity SpCas9 variants

Supplementary Figure 2. Off-target analysis for Cas9-NG, SpG and SpRY

Supplementary Figure 3. SpRY off-target at NNN PAM

Supplementary Figure 4. Statistics for translocations and large deletions for SpCas9 variants at NGG sites

Supplementary Figure 5. Self-targeting for SpCas9-flexible variants

Supplementary Figure 6. Summary of the engineered SpRY variants mutation site

Supplementary Table. Detected off-target site for all SpCas9 variants, primer and gRNA information for PEM-seq

Supplementary Figure1

A

SpCas9 variants	Mutation information
eCas9	K848A K1003A R1060A
HF1	N497A R661A Q695A Q926A
FeCas9	K848A K1003A R1060A D1135E
evoCas9	M495V Y515N K526E R661Q
HiFi	R691A
HypaCas9	N692A M694A Q695A H698A
LZ3	N690C T769I G915M N980K
Sniper	F539S M763I K890N
Cas9-NG	R1335V L111R D1135V G1218R E1219F A1322R T1337R
xCas9	A262T R324L S409I E480K E543D M694I E1219V
SpG	D1135L S1136W G1218K E1219Q R1335Q T1337R
SpRY	SpG (L1111R/A1322R) + A61R N1317R R1333P

B

OT	Sequence
RAG1	GCCTCTTTCCCACCCACCTT GGG
OT1	TCCTCCTCCCCACCCACCTT CAG
OT2	ACCCCTTCCCCACCTACCTT GGG
OT3	CCCTCCTTCCCACCCACTTT GGG
OT4	ACTTCCTTCCCACCCACCTT CAG
OT5	ACGTCTTCCCCACCCACCTG GGG
OT6	TCCTCTCTCCCACCCACCTC CGG
OT7	CACCCTTTCCCATCCACCTT TGG
OT8	GTCTCTTTCTCACCCACTTT GGG
OT9	TCCTCTTCTCCACCCACCAT AGG
OT10	TTCTCTTCCCCACCCACCTT TGA
OT11	GCCTCTTCCACACCCACCCT GGG
OT12	TCCTCTTCTCCACCCACCTC TGG
OT13	GCCTCTTTACCACCTCACCT TGG
OT14	GACTCTTTTCCACCCACCCT AGG
OT15	TGCTCTTCCCCACCCACCCA AGG
OT16	GTCTCTTTCCCATCCACCTT TGA
OT17	GTCTCTTCCTCACCCACCTG GGC
OT18	GCCCCTACCCCACCCACCTG GGT
OT19	GCCCCTCCCCCACCCACCCT GGG
OT20	ACCTCCTTCCCGCCCACCTG GGG
OT21	TTTTCTTCCCCACCCACCTC GGG
OT22	ACCTCTTATCCACCCACCTT GGC
OT23	GCCCCTTCCCCACCCACCCT GTG

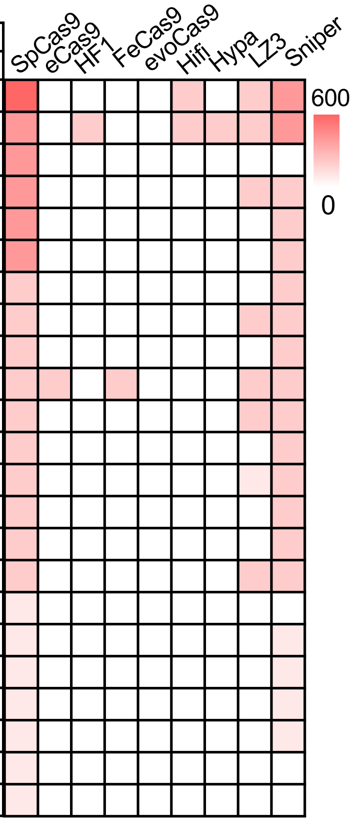

C "Trade-off" for SpCas9 high-fidelity variants

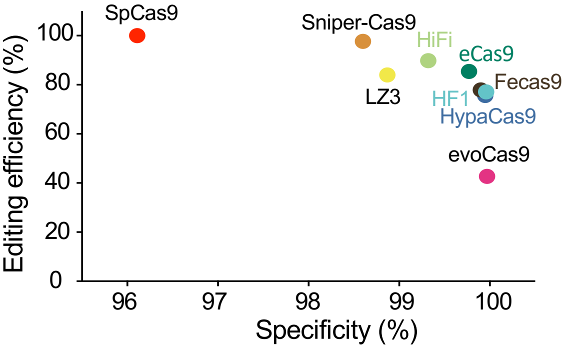

D

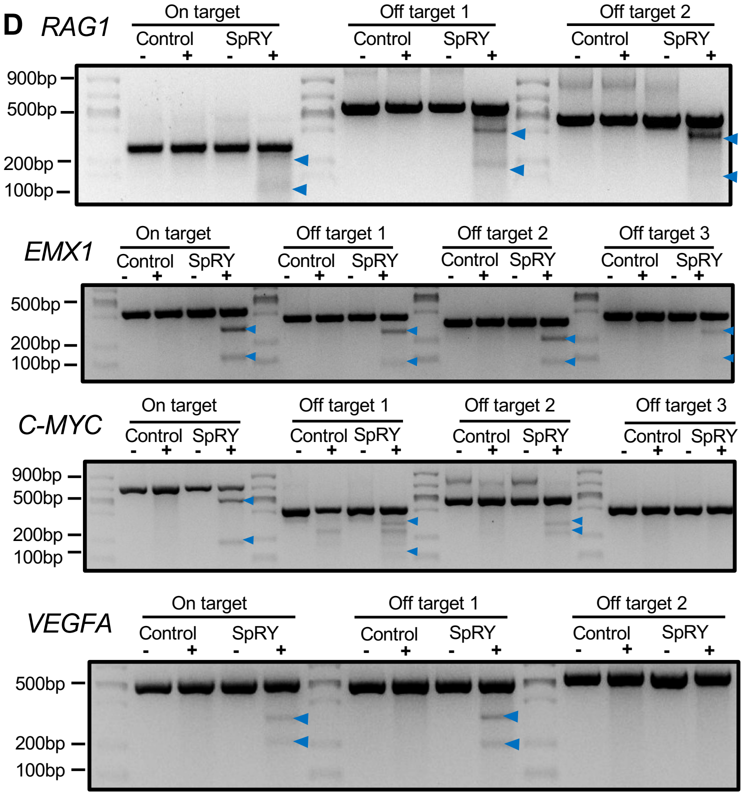

E

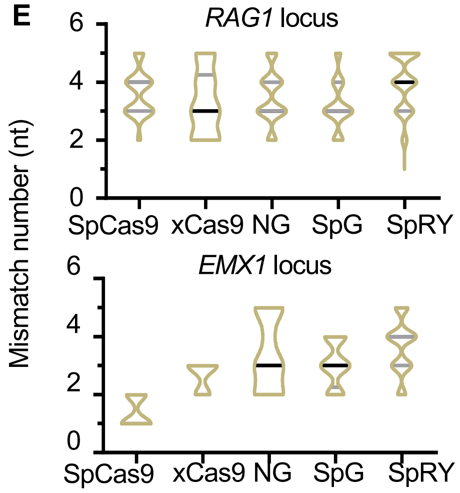

F

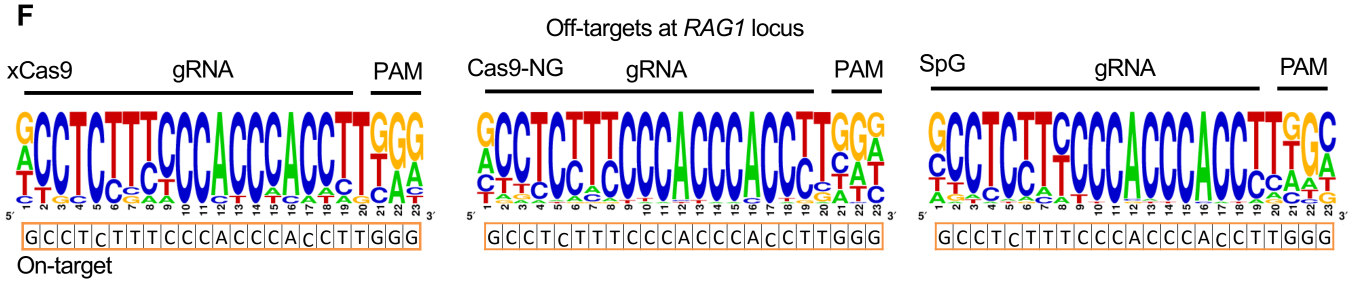

Supplementary Figure 1. Evaluation of the editing efficiency and specificity for 8 high-fidelity SpCas9 variants. **(A)** Summary of mutations of all the tested SpCas9 variants.**(B)** Off-target sequence and relative cutting frequencies at *RAG1* locus for all high-fidelity variants detected by PEM-seq. Left: off-target sequence. The mismatched nucleotides with the target site are highlighted in red. Right: A heat map showing the off-target density. **(C)** 2D visualization for the editing efficiency and specificity of eight high-fidelity SpCas9 variants. The editing efficiency and specificity are the mean of five NGG target loci. Specificity is calculated as 1- (percentage of off target junctions normalized to editing events). **(D)** T7 endonuclease I (T7EI) assay for the top 2-3 off-target of SpRY at four NGG loci (*RAG1, C-MYC, EMX1, VEGFA*). The cleavage strand is indicated with blue arrows. **(E)** gRNA mismatch numbers of *RAG1* and *EMX1* off-targets for SpCas9 and PAM-flexible variants. **(F)** Consensus sequence analysis for xCas9, Cas9-NG and SpG off-targets at *RAG1* locus detected by PEM-seq. On-target sequence is marked below and position for gRNA and PAM is labelled above.

Supplementary Figure 2

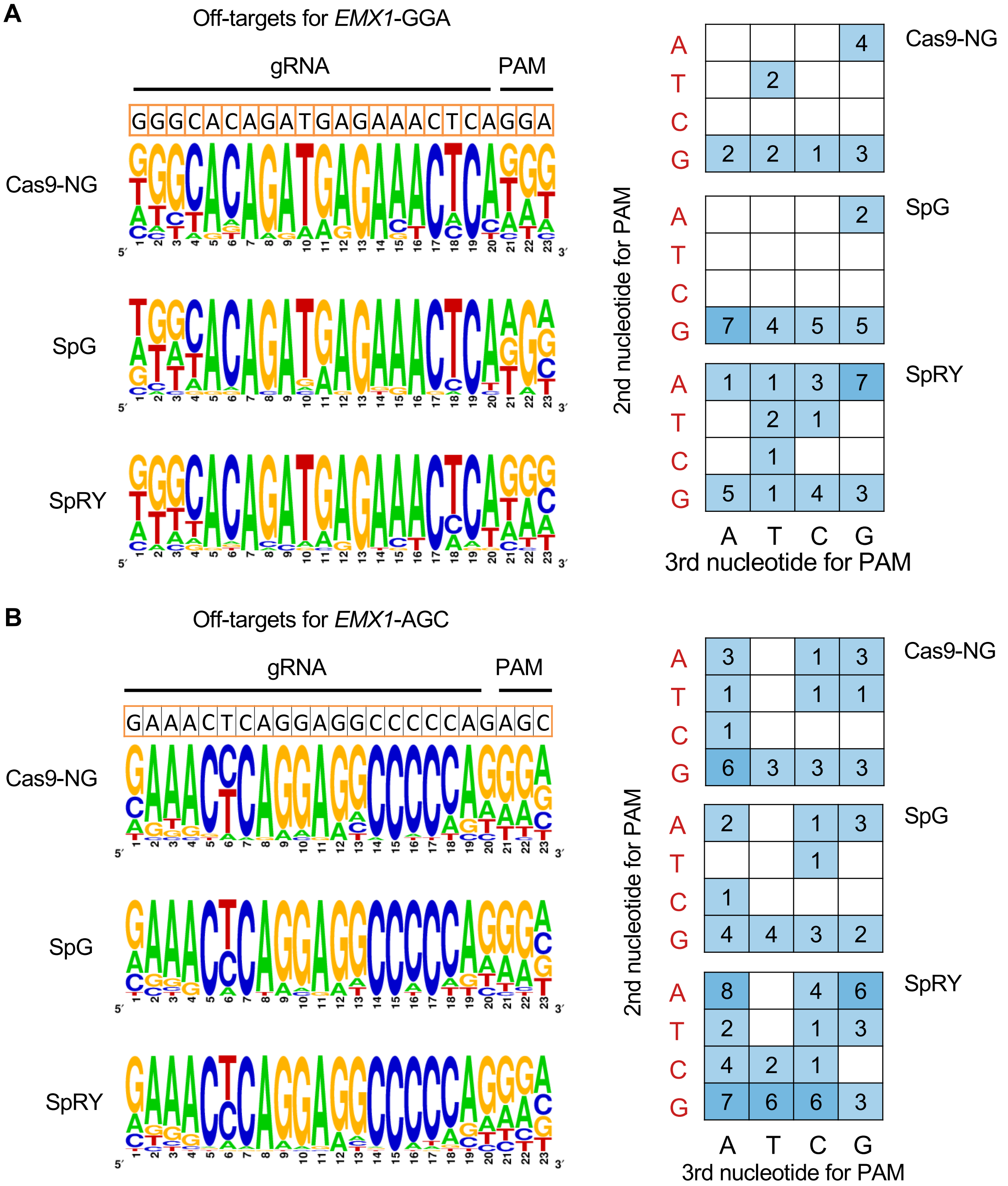

Supplementary Figure 2. Off-target analysis for Cas9-NG, SpG and SpRY. (A and B) Left: Weblogo showing consensus off-target sequence of xCas9, Cas9-NG and SpRY at **(A)** *EMX1*-GGA and **(B)** *EMX1*-AGC loci. Sequence in the orange box indicated the on-target. Right: analysis for the 2nd and 3rd nucleotides of PAM sequence of Cas9-NG, SpG and SpRY at **(A)** *EMX1*-GGA and **(B)** *EMX1*-AGC loci. Numbers in the heatmap box indicate the off-target counts. The 2nd nucleotide for PAM is highlighted in red.

Supplementary Figure 3

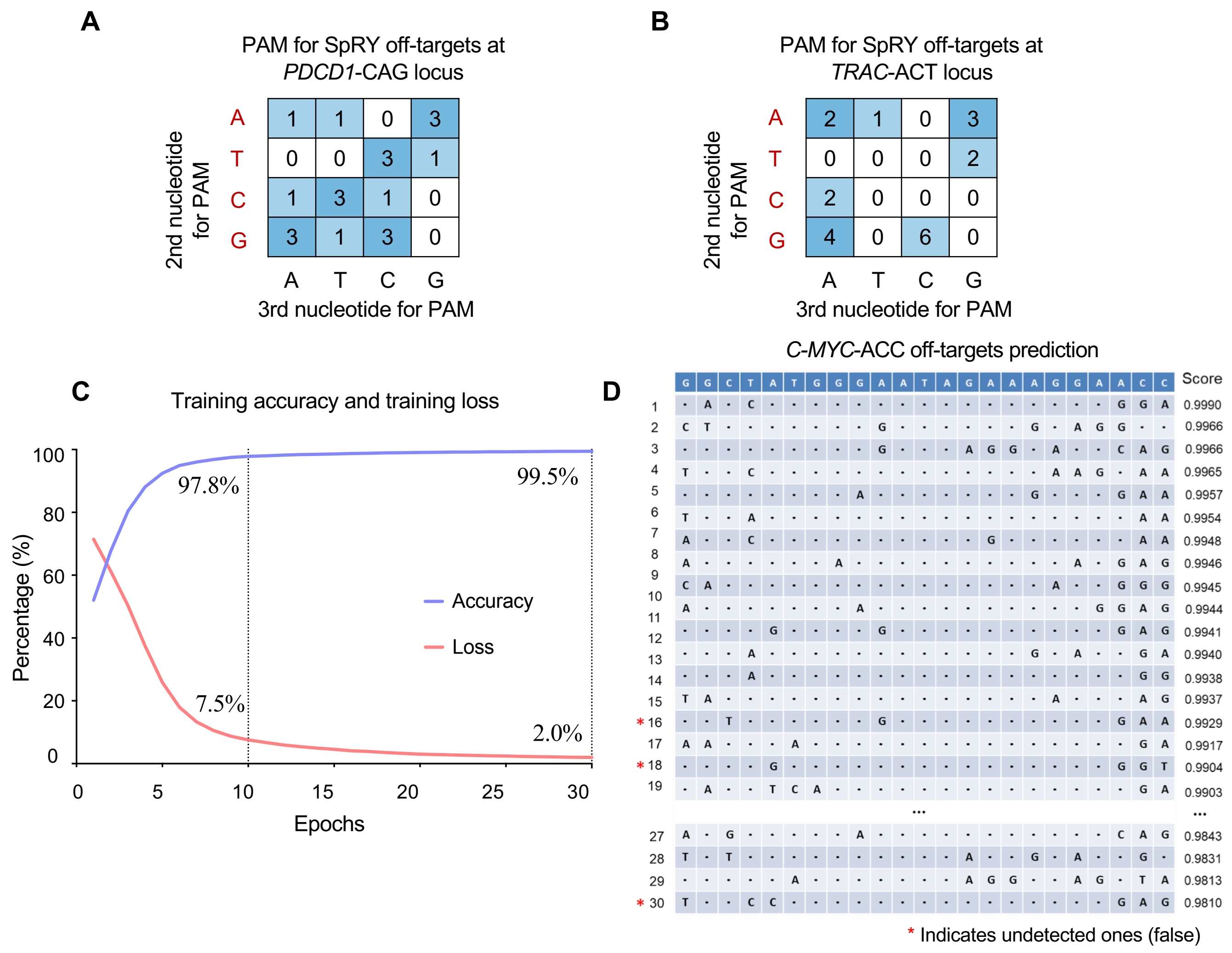

Supplementary Figure 3. SpRY off-target at NNN PAM. (A and B) Statistics for the 2nd and 3rd nucleotides of PAM for SpRY off-targets at **(A)** *PDCD1*-CAG and **(B)** *TRAC*-ACT loci. Numbers in the heatmap box indicate the off-target counts. The 2nd nucleotide sequence is highlighted in red. **(C)** The accuracy and loss rate along with training epochs. The accuracy and loss rates are marked at the 10th and 30th epochs. **(D)** Top 30 off-targets predicted by deep learning model for *C-MYC*-ACC locus. The 3 false off-targets are indicated by *. Prediction scores are on the right.

Supplementary Figure 4

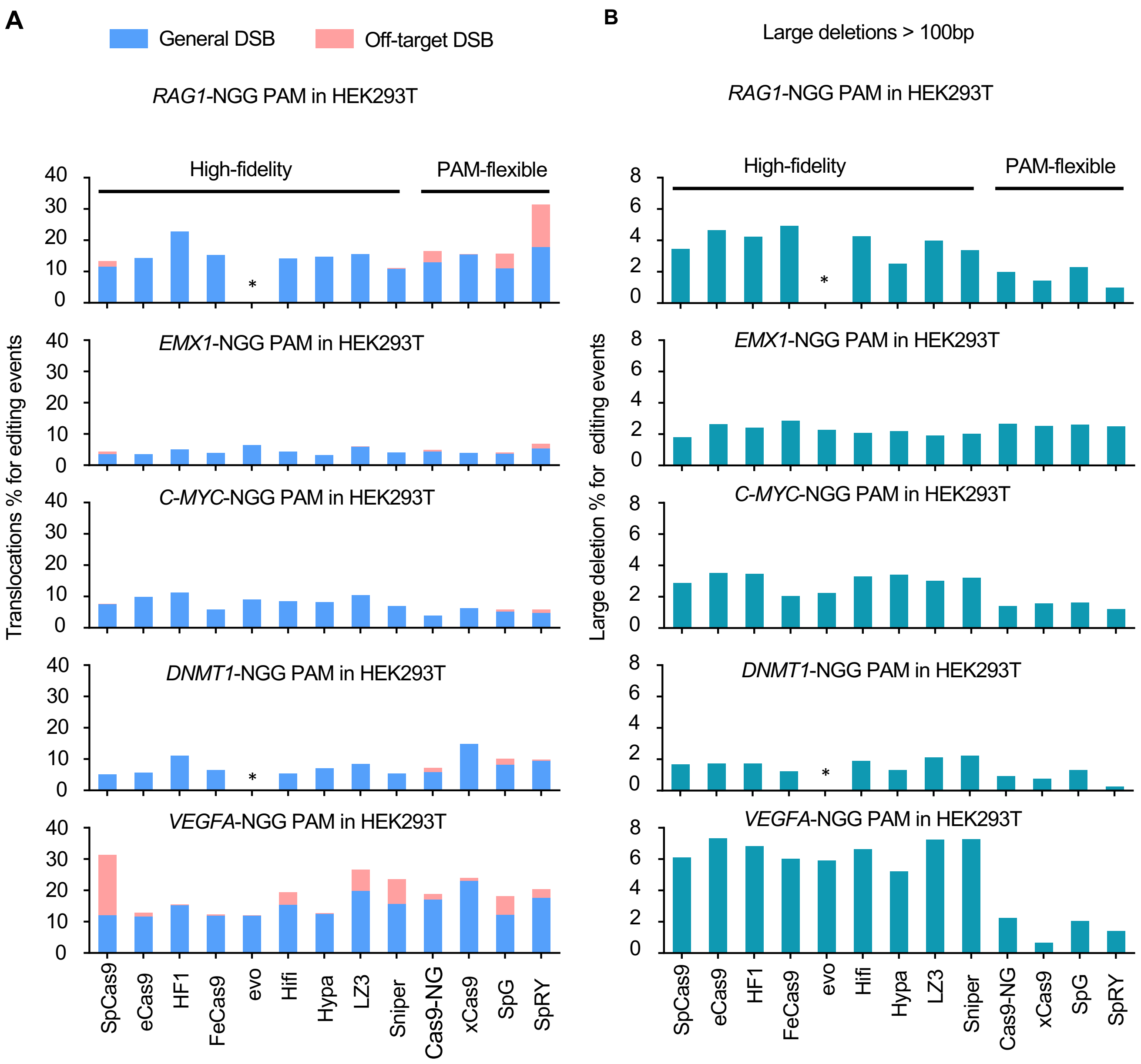

Supplementary Figure 4. Statistics for translocations and large deletions for SpCas9 variants at NGG sites. (A) Percentages of translocation normalized to editing events of indicated SpCas9 and variants at *RAG1, EMX1, C-MYC, DNMT1, VEGFA* loci detected by PEM-seq. Blue bars indicate chromosomal translocation with general DSBs and pink with off-target DSBs. **(B)** Percentages of large deletion (> 100bp) normalized to editing events for indicated SpCas9 and SpCas9 variants at *RAG1, EMX1, C-MYC, DNMT1, VEGFA* loci detected by PEM-seq. * represents nearly no editing efficiency at this locus.

Supplementary Figure 5

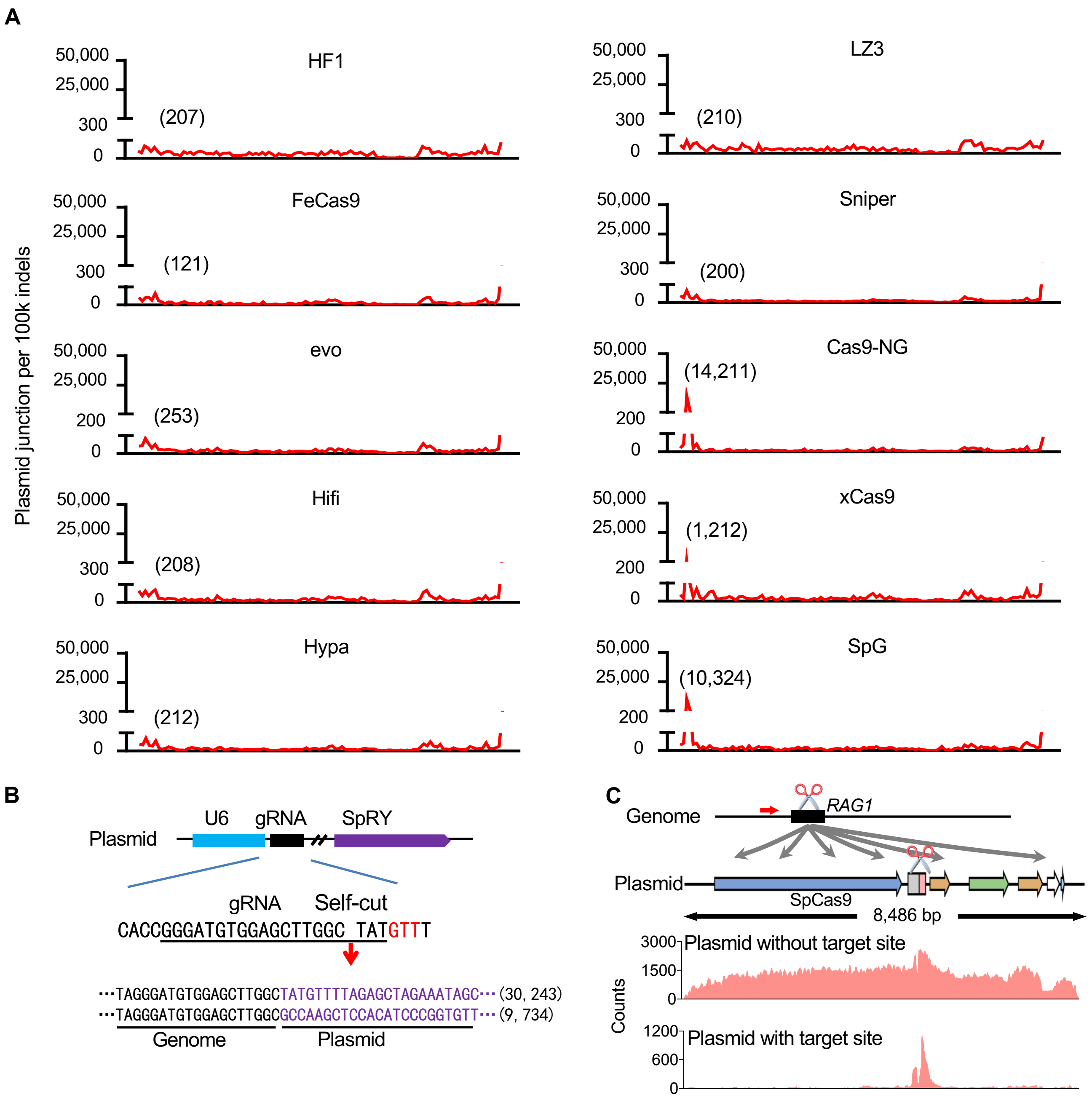

Supplementary Figure 5. Self-targeting for SpCas9 -flexible variants. **(A)** The distribution of plasmid junctions across the plasmid backbone every 100k indels for other SpCas9 variants at indicated NGG loci in HEK293T cells detected by PEM-seq. **(B)** The numbers of direct translocation junctions between genomic target sites and the two plasmid broken ends at the *C-MYC* locus . "GGGATGTGGAGCTTGGC TAT" indicates *C-MYC* gRNA sequence. The sequences of translocation junctions are showed with genomic sequence in black and plasmid sequence in purple. Numbers are showed on the right. **(C)** Distribution for plasmid junctions detected by PEM-seq cloning from *RAG1* cleaved locus in the genome. The bottom orange-filled plot indicates plasmid junctions detected by the PEM-seq with an extra *bona fide RAG1* target site in the plasmid. Black box represents the *RAG1* gene in the genome and the red arrow indicates the position and orientation of primer used for PEM-seq. Grey arrows represents translocation between the genome and the plasmid. The scissors indicate the cleavage site of SpCas9 in the plasmid.

Supplementary Figure 6

Mutations relative to SpCas9

	61	497	661	691	692	694	695	698	848	926	1003	1060	1111	1135	1136	1218	1219	1317	1322	1333	1335	1337
SpCas9	A	N	R	R	N	M	Q	H	K	Q	K	R	L	D	S	G	E	N	A	R	R	T
Sp*RY*	R												R	L	W	K	Q	R	R	P	Q	R
HF1-SpRY	R	A	A				A			A			R	L	W	K	Q	R	R	P	Q	R
eCas9-SpRY	R								A		A	A	R	L	W	K	Q	R	R	P	Q	R
Hypa-SpRY	R				A	A	A	A					R	L	W	K	Q	R	R	P	Q	R

Supplementary Figure 6. Summary of the engineered SpRY variants mutation site. The sites for mutated amino acids are shown on the top and the original amino acids are presented in the SpCas9 line. Corresponding mutations are indicated in the table for SpRY and three new SpCas9 variants.
